# Advances and Challenges in Orthopedic Implant-Associated Infections

**DOI:** 10.3390/healthcare12181819

**Published:** 2024-09-11

**Authors:** Olimpio Galasso, Alessandro Crinisio, Alessandro Bartoli, Biagio Moretti

**Affiliations:** 1 Department of Medicine, Surgery and Dentistry, University of Salerno, 84081 Salerno, Italy; alessandrocrinisio@gmail.com (A.C.); a.bartoli1@studenti.unisa.it (A.B.); 2 Orthopedic Department “Clinica Ortopedica”, San Giovanni di Dio and Ruggi d’Aragona University Hospital, 88100 Catanzaro, Italy; 3 Orthopaedic and Trauma Unit, Department DiBraiN, University of Bari “Aldo Moro”, 70124 Bari, Italy; biagio.moretti@uniba.it

The number for orthopedic implants and reconstructive joint replacements, including knee and hip implants, underwent a considerable increase, driven by the aging population and increasing prevalence of musculoskeletal disorders. Orthopedic implant-associated infection remains a significant concern, potentially leading to severe complications, including prolonged hospital stays, increased healthcare costs, and potentially devastating outcomes for patients. The incidence of periprosthetic infections is highest in the first two years after surgery, and women are at higher risk than men [1].

The management of infections in orthopedics represents a formidable challenge, demanding vigilance, innovation, and collaboration from the medical community [2]. Prevention of infection and the choice for surgical indication are essential to minimize the risk of orthopedic implant-associated infections.

The articles collected in this Special Issue have evidenced this: the identification of certain risk factors, the introduction of rigorous diagnostic protocols and new promising laboratory tests, as well as the development of advanced surgical techniques and effective antimicrobial agents, have reduced overall infection rates.

Factors that increase the risk of infection include patient-related factors such as advanced age, diabetes, obesity, smoking, immunosuppression, and malnutrition and procedure-related factors such as length of surgery, use of prosthetic implants, and the complexity of the surgical procedure.

Research has been directed towards the utilization of inflammatory biomarkers for early detection of complications in hip and knee replacement surgeries [3]. In the context of orthopedic implant-associated infections, CRP is particularly significant for acute infection, where an immediate postoperative increase in the CRP level is detected; however, the diagnosis of a subacute or chronic infection remains a greater challenge. Combining serological test results can improve diagnostic accuracy since definitive conclusions cannot be drawn from a single diagnostic test [4]. New diagnostic criteria have been introduced to address the limitations of the prior definitions [5]. The new criteria and introduction of novel tests have helped to improve diagnostic accuracy, showing a 97.7% sensitivity and 99.5% specificity.

Effective management of PJI requires a combination of medical and surgical approaches tailored to the individual patient’s situation. Antibiotic selection depends on several factors, such as the organism that is identified and its antibiotic resistance profile, the extent of the infection, and factors related to the patient [6]. Major surgical strategies for the treatment of PJI include debridement, antibiotic, and implant retention (DAIR) and debridement, antibiotic pearls, and retention of the implant (DAPRI) [7,8], one-stage or two-stage implant replacement [9,10]. Resection arthroplasty with no reimplantation, or amputation represent surgical options in patients with severe comorbidities or failed previous revisions [11]. The choice of surgical approach is based on data from an increasing number of experimental and cohort studies.

Despite this knowledge, orthopedic infections continue to pose serious risks. The increasing prevalence of antibiotic-resistant bacteria, the complexities of treating periprosthetic joint infections, and the challenges associated with biofilm formation on implants underscore the need for ongoing research and innovation. It is crucial to stay ahead of these evolving pathogens through continuous surveillance, research, and the development of new therapeutic strategies. However, prevention remains the cornerstone of managing orthopedic infections [12]. Emphasizing preoperative optimization, meticulous surgical techniques, and postoperative care can significantly reduce infection rates. Additionally, the role of patient education cannot be overstated; informed patients are more likely to adhere to pre- and post-operative guidelines, thereby reducing their risk of infection [13].
